# Dynamic Neural Network States During Social and Non-Social Cueing in Virtual Reality Working Memory Tasks: A Leading Eigenvector Dynamics Analysis Approach

**DOI:** 10.3390/brainsci15010004

**Published:** 2024-12-24

**Authors:** Pinar Ozel

**Affiliations:** Electric and Electronic Engineering Department, Istanbul University-Cerrahpasa, Istanbul 34320, Turkey; pozell@gmail.com

**Keywords:** dynamic brain connectivity, EEG, LEiDA, social cues, virtual reality, deep learning

## Abstract

Background/Objectives: This research investigates brain connectivity patterns in reaction to social and non-social stimuli within a virtual reality environment, emphasizing their impact on cognitive functions, specifically working memory. Methods: Employing the LEiDA framework with EEG data from 47 participants, I examined dynamic brain network states elicited by social avatars compared to non-social stick cues during a VR memory task. Through the integration of LEiDA with deep learning and graph theory analyses, unique connectivity patterns associated with cue type were discerned, underscoring the substantial influence of social cues on cognitive processes. LEiDA, conventionally utilized with fMRI, was creatively employed in EEG to detect swift alterations in brain network states, offering insights into cognitive processing dynamics. Results: The findings indicate distinct neural states for social and non-social cues; notably, social cues correlated with a unique brain state characterized by increased connectivity within self-referential and memory-processing networks, implying greater cognitive engagement. Moreover, deep learning attained approximately 99% accuracy in differentiating cue contexts, highlighting the efficacy of prominent eigenvectors from LEiDA in EEG analysis. Analysis of graph theory also uncovered structural network disparities, signifying enhanced integration in contexts involving social cues. Conclusions: This multi-method approach elucidates the dynamic influence of social cues on brain connectivity and cognition, establishing a basis for VR-based cognitive rehabilitation and immersive learning, wherein social signals may significantly enhance cognitive function.

## 1. Introduction

In recent years, the combination of neuroimaging and virtual reality (VR) has provided new opportunities for studying the brain dynamics of cognitive processes [1]. Working memory, an essential cognitive function in daily tasks, has been widely examined using conventional methods [2]. However, the emergence of VR technology offers a distinct opportunity to replicate real-life situations that involve both social and non-social cognitive processes [3]. Using electroencephalography (EEG) to measure neural activity during these tasks provides detailed and precise information regarding the brain states involved in these cognitive processes [4].

Research on the neural correlates of social and non-social cues has uncovered divergent patterns of cerebral activity. One study [5] investigates the impact of repetitive transcranial magnetic stimulation on functional connectivity in methamphetamine use disorder, offering insights into neural adaptations. The neural mechanisms underlying social contextual influences on adolescent risk-taking behavior have been examined, enhancing our comprehension of how intricate social environments affect neural responses. This is detailed in a study presented by [6].

VR is an appropriate platform for studying complicated cognitive tasks because it incorporates real-world features and allows for controlled modification of environmental stimuli [7]. The distinction between social and non-social cueing in VR can provide insight into how human cognition adjusts in various social situations, an area that has received less attention in traditional cognitive neuroscience [8]. Research has demonstrated that social interactions in VR can have a notable impact on cognitive load and the corresponding brain reactions [9]. This reveals the existence of separate neural networks that are activated during social activities compared with non-social tasks [10,11].

Studies have shown that VR interventions can improve social skills and emotional recognition in children and adolescents, including those with neurological disorders. One study [12] demonstrates the efficacy of VR in enhancing social and emotional learning. The influence of haptic feedback on social interactions in VR has been investigated, demonstrating its effect on user experience and the genuineness of social touch. Ref. [13] elucidates the influence of tactile feedback on social dynamics in virtual environments. The importance of social cues in mediated, bidirectional, multiparty interactions has been examined, highlighting their function in enhancing interpersonal communication. A study published in 2021 [14] examines the significance of different social cues in immersive environments.

VR has become a revolutionary instrument in multiple domains, offering immersive and interactive settings that replicate real-world situations. One of its most significant applications is in the medical field, where avatars in VR environments are employed for therapeutic and training objectives. VR interventions have demonstrated efficacy in treating mental health disorders, including social anxiety, post-traumatic stress disorder (PTSD), and depression, by enabling patients to interact with regulated virtual environments [15,16]. Moreover, avatars in VR are extensively utilized for cognitive rehabilitation, aiding patients in the re-acquisition of motor and cognitive skills after neurological injuries [17]. This research utilizes the distinctive features of VR to examine dynamic brain network states during interactions with social avatars.

Connectivity analysis grounded in graph theory provides critical insights into the structural organization and complexity of brain networks by depicting brain regions as nodes and significant functional connections as edges. This method is especially beneficial for examining dynamic brain states, as it facilitates the analysis of network characteristics, including the clustering coefficient and path length, which enhance comprehension of the neural mechanisms that underpin cognitive functions. Utilizing graph theory on EEG-derived coherence matrices allows for the examination of connectivity patterns across various cognitive states, especially in VR environments where social and non-social stimuli may elicit distinct network configurations [18,19,20].

Dynamic functional connectivity (DFC) refers to temporary and state-specific changes in connections between different brain networks [21]. It is considered an important factor in cognitive function. The high temporal resolution (TR) of EEG is particularly suitable for this type of analysis [22,23]. Some common DFC methods used with EEG are time–frequency resolution coherence analysis, phase synchronization analysis, sliding-window correlation analysis, graph-theory-based methods, and wavelet coherence. Time–frequency analysis was used to analyze the power and coherence of the EEG signals in different frequency bands. This method makes it possible to monitor coherence changes in signals over time and determine how synchronization between brain regions changes during specific tasks or sensory stimulation [24]. Phase synchronization analysis measures the extent to which the phases of EEG signals from different brain regions are in harmony. Phase synchronization is used to examine temporal connections and collaborations between neural networks [25]. The phase locking value (PLV) is a measure used to quantify the consistency of phase differences between signals over time [26]. Sliding-window correlation analysis continuously calculates the correlations between EEG signals using a specified window size. The window is shifted across the signal, revealing connectivity structures that change over time. This method is particularly suitable for tracking short-term changes in brain dynamics [27]. Moreover, EEG data can be analyzed using graph theory, in which brain regions are represented as nodes and the connectivity between them as edges. This approach helps us to understand the structural and functional properties of brain networks by evaluating their topological properties (e.g., the clustering coefficient and path length) [28]. Wavelet coherence measures the coherence between two signals at different time points and in different frequency bands, thereby enabling a more detailed dynamic connectivity analysis [29]. Progress in EEG data analysis has yielded novel techniques for evaluating functional connectivity [30]. A thorough review [31] examines contemporary methods for EEG seizure detection, emphasizing the challenges and prospective advancements in the domain. Furthermore, the amalgamation of EEG and ensemble learning has been suggested for precise grading and staging of diseases, highlighting the essential significance of time window length in assessing model stability and efficacy. This methodology is elucidated in a study [32]. The utilization of graph convolutional networks (GCNs) for processing graph-structured EEG data has demonstrated potential in patient-independent epileptic seizure detection, as outlined in [33].

Recent progress in DFC analysis approaches provides an exceptional opportunity to investigate these dynamics with very detailed spatial and temporal resolutions. The recently established Leading Eigenvector Dynamics Analysis (LEiDA) method has played a crucial role in capturing these changes by examining the patterns of leading eigenvectors derived from connectivity matrices [34]. This method offers a new way to observe changes [20] in neural network states linked to various cognitive demands.

The LEiDA method was developed to monitor the functional connectivity changes in the brain over time. This method is used specifically with functional magnetic resonance imaging (fMRI) data because fMRI measures changes in cerebral blood flow to visualize brain activity, which can reflect dynamic connectivity changes in the brain over long-term periods [35,36,37]. LEiDA analyzes the dynamics of leading eigenvectors in time-series data obtained from fMRI. These eigenvectors represent the strongest connectivity patterns in the brain at a given time point and show how these connections change over time. This method is particularly useful for understanding how brain networks organize and reorganize during complex cognitive processes [38,39].

The use of LEiDA with EEG is a newer field of research. EEG records brain waves with a high TR at the millisecond level, which provides the opportunity to observe much faster brain dynamics [40]. However, EEG has a lower spatial resolution than fMRI, which may limit some types of analysis [41]. The use of methods such as LEiDA combined with EEG data offers the potential to examine time-varying brain network dynamics in more detail. In particular, the high TR of EEG, when combined with the LEiDA method, can capture rapid changes in brain networks and allow more sensitive examination of cognitive processes. Additionally, Zhou et al. stated in their study that LEiDA has a superior TR compared to other DFC analysis methods, such as the sliding-window method [42]. Therefore, although the LEiDA method is mostly used with fMRI data, it can also be used with EEG data, and this combination can open new doors for a more comprehensive understanding of brain dynamics. Such an approach could provide great advantages, especially in studies on rapidly changing cognitive states and responses.

The term ‘states’ specifically denotes dynamic brain network states identified via LEiDA. These states signify persistent patterns of phase synchronization among various brain regions, as calculated from the dynamic phase-locking matrices (d) obtained from EEG data. Each state represents a distinct configuration of functional connectivity, reflecting the temporal dynamics of brain network organization throughout the task [34]. The identification of these states enables an examination of how the brain transitions between various connectivity patterns during interactions with social and non-social cues. In this study, the term PL states is used to refer to the states identified by LEiDA.

This research examines a significant deficiency in comprehending the dynamic neural mechanisms that underpin social cognition, especially during real-time interactions. Conventional investigations of brain connectivity typically depend on static analyses that average connectivity patterns over time, thereby neglecting the transient and rapidly changing states of brain networks during social interactions. This constraint is particularly problematic when examining intricate cognitive processes such as social cueing, which necessitate temporal precision to comprehend how the brain shifts between various functional states. This research employs the LEiDA method to address these challenges by providing a high-resolution, temporally sensitive framework for examining brain network dynamics.

This study incorporates an additional innovative element through the utilization of VR. In contrast to traditional experimental configurations, VR offers an immersive and ecologically valid environment for simulating authentic social interactions. This enables the examination of neural responses in an environment that closely mirrors natural social contexts, providing enhanced understanding of the mechanisms underlying social cognition. The utilization of VR to examine dynamic network states in reaction to social and non-social stimuli is both timely and innovative, as it connects laboratory research with the intricacies of real-world interactions.

LEiDA is particularly adept for this function as it directly examines the temporal dynamics of phase-locking matrices, discerning recurrent connectivity patterns with elevated temporal resolution. In contrast to sliding-window correlation (SWC), which is constrained by arbitrary window size and diminished sensitivity to swift transitions, LEiDA effectively captures nuanced, instantaneous alterations in brain connectivity. Likewise, although graph theory offers significant structural insights into brain networks, its metrics are frequently based on static representations and are incapable of analyzing temporal transitions. By integrating LEiDA with VR, I seek to offer a sophisticated comprehension of the brain’s dynamic processing of social information in real time.

A primary impetus for this research is the opportunity to create targeted therapeutic interventions for disorders marked by impairments in social cognition, including autism spectrum disorder, schizophrenia, and social anxiety. Contemporary therapeutic approaches frequently neglect the dynamic characteristics of social interactions, prioritizing static or excessively simplistic models instead. By pinpointing particular brain network states linked to social and non-social interactions, my results may guide the development of VR-based interventions aimed at enhancing individuals’ social functioning within a controlled yet realistic setting.

This research incorporates sophisticated analytical techniques, such as deep learning and graph theory, to enhance the LEiDA methodology. Deep learning facilitates the identification of complex patterns in high-resolution EEG data, revealing nuanced dynamics that conventional methods may overlook. Graph theory provides a structural perspective, illustrating how network attributes such as clustering coefficients and path lengths vary with different cognitive tasks. The amalgamation of these methodologies offers a thorough framework for comprehending the temporal and structural dimensions of brain network dynamics, facilitating a comprehensive exploration of cognitive processes.

This study enhances our comprehension of social cognition at the neural level by examining the transitions between dynamic brain network states during social and non-social cueing in VR. It also tackles the overarching challenge in neuroscience of examining brain networks in genuine, ecologically valid environments. This research addresses a significant gap in the literature and establishes a foundation for novel therapeutic strategies designed for practical social deficits, providing valuable applications for clinical populations [34,43].

## 2. Materials and Methods

**Figure 1:**
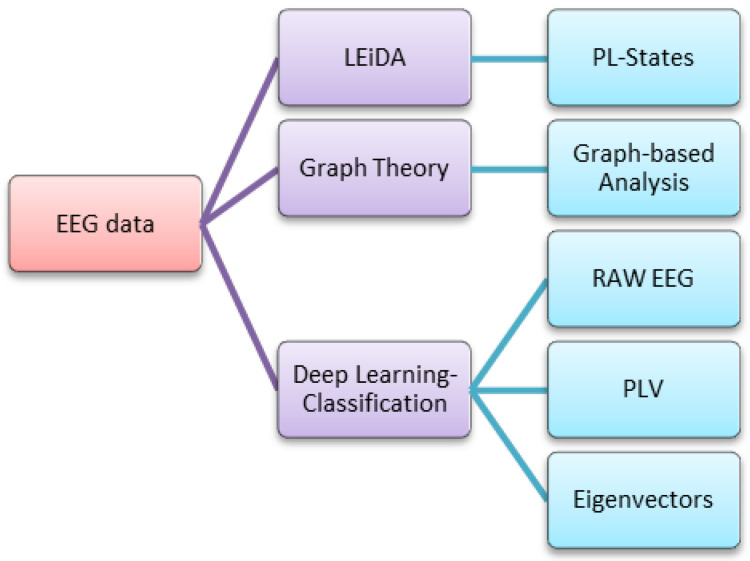
Applied methods (LEiDA, graph theory, and deep learning classification).

In this study, three main themes were examined, namely LEiDA, graph theory, and deep learning. LEiDA analysis (Section 2.2) was performed to obtain PL states in the brain. In the graph theory part (Section 2.3), graph-based analysis was performed using different graphs. In the deep learning section (Section 2.4), classification results were evaluated using raw EEG, PLVs, and Eigenvectors. The methods applied are visualized in Figure 1.

### 2.1. Dataset

#### 2.1.1. Participant Demographics

This study utilized a dataset originally published by [44] comprising EEG recordings obtained during a VR working memory task involving the presentation of social and non-social cues to participants. The dataset was released to the public to facilitate research on cognitive processes within VR environments.

This research included 47 participants (25 females and 22 males) from a university community, all with normal or corrected-to-normal vision and no history of neurological or psychiatric disorders. Their average age was 24.3 years (SD = 4.7). Informed consent was obtained from all participants, and the study was approved by the institutional review board.

Although a formal power analysis to justify the sample size of 47 participants could not be performed due to time constraints, this limitation is addressed by referencing similar studies in the field that utilized a comparable number of participants. These references provide context and support for the adequacy of the sample size in this research [12,32].

#### 2.1.2. VR Working Memory Task

**Figure 2:**
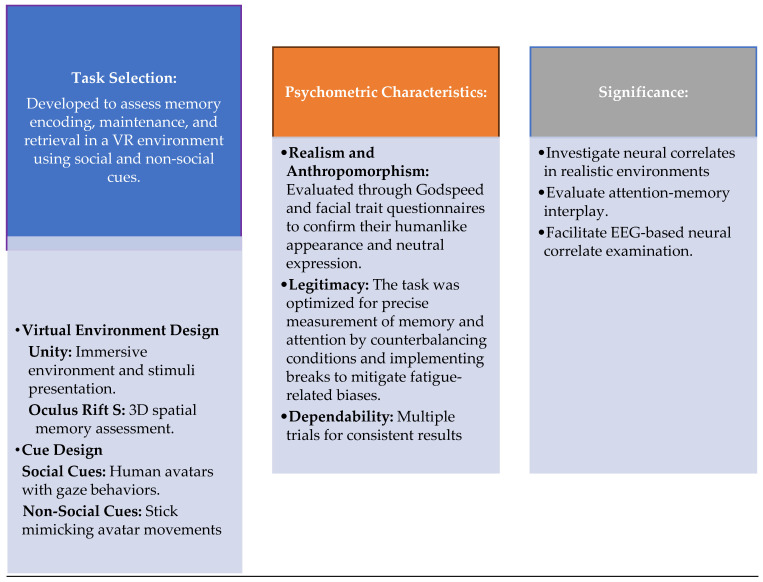
VR working memory task: selection and design schema.

Participants were immersed in a VR environment using a head-mounted display. The task assessed working memory through memorization of the location and characteristics of the objects displayed on a virtual table. Two types of attentional cues were utilized: social avatars (e.g., a virtual character making eye contact and pointing) and non-social stick cues (e.g., a stick arrow pointing). The task structure included multiple trials, each with cueing, memorization, and recall phases. The object details, participant responses, and reaction times were recorded for each trial. The criteria used to select the VR working memory task and its psychometric properties are detailed in reference [44]. This process is summarized in Figure 2 for this study.

#### 2.1.3. Experiment Procedure

**Figure 3:**
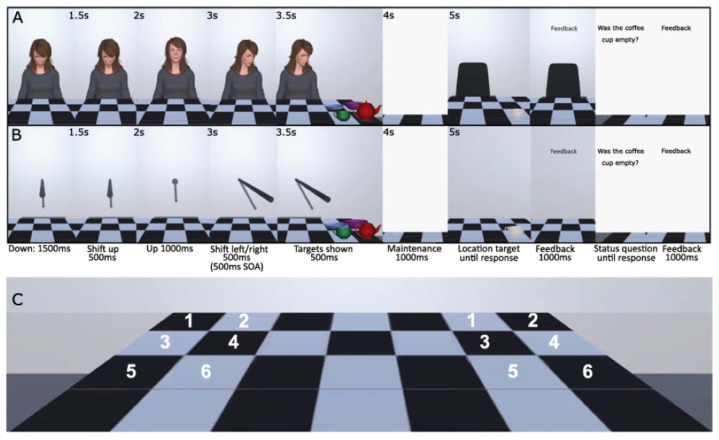
Depiction of the trial process (checkered pattern inspired by [45]). Utilizing the parameters of the conventional central cueing paradigm, the cue persisted on the screen for the duration of the trial (e.g., [46,47]). Panel (**A**) shows the social avatar cue, and Panel (**B**) shows the non-social stick cue. Timings, as depicted in the figure, were synchronized across cue types. The inter-trial interval was 1000 ms, during which a fixation cross was displayed. The experiment was a free-viewing study, allowing participants to move their eyes freely. Panel (**C**) shows the six possible left and right locations for the four encoding targets.

Using a head-mounted display, 3D avatars engaged participants by making eye contact before looking towards the intended object location, mimicking real eye movement behaviors. Target items—kitchen utensils such as bowls, plates, cups, and teapots—appeared on either side of a table, facilitating the study of cue influence in a realistic setting. Participants memorized multidimensional information about items, such as location and status, during the cue shift, encoding, and retrieval phases. A dynamic 3D stick served as a non-social control cue, paralleling the movement of social avatars (Figure 3).

#### 2.1.4. Apparatus and EEG Acquisition

The experiment was programmed in Unity and was conducted using a Lenovo Legion Y540-17IRH laptop (Beijing, China) with an Oculus Rift S PC-Powered VR Gaming Head-Mounted Display. EEG data were captured using a 64-channel ANT Neuro eego™ (Hengelo, The Netherlands) sports mobile EEG system following the international 10–20 electrode placement system. The data sampling rate was 500 Hz with online filtering between 0.1 and 100 Hz. Eye movements and blinks were monitored using additional electrodes. The preprocessing steps included band-pass filtering (1–40 Hz), artifact rejection, and independent component analysis (ICA) for correction of eye movement and muscle artifacts [44].

### 2.2. LEiDA Analysis

**Figure 4:**
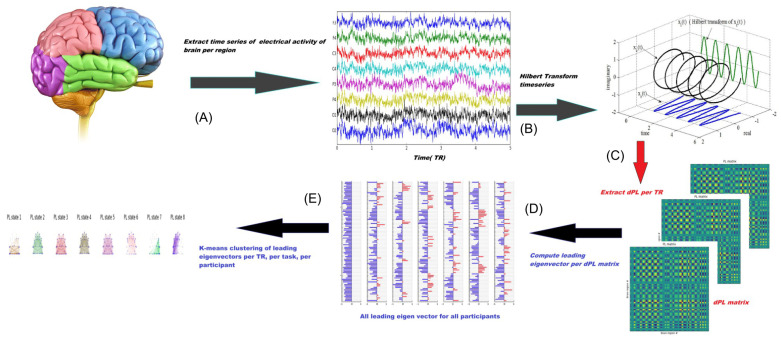
Extraction of EEG signal PL states. (**A**) For a given region, the EEG signal is first preprocessed. (**B**) Hilbert transformation is applied in order to acquire an analytic signal, whose phase can be represented over time and each TR (temporal resolution), which refers to the time interval between consecutive data samples, utilized for monitoring dynamic connectivity alterations. (**C**) The dPL(t) matrix quantifies the degree of phase synchronization between each pair of areas. The dominant eigenvector of the dPL(t) matrix, denoted as V(t), represents the primary direction of all phases. Each element in V(t) corresponds to the projection of the phase of each region onto V(t) (right). (**D**) The eigenvectors V(t) from all participants are combined and inputted into a k-means clustering algorithm, which separates the data points into a predetermined number of groups, k. (**E**) Every cluster centroid symbolizes a recurring PL state. dPL refers to dynamic phase-locking (Enhancing Clarity: Process Summary {1. Preprocessing →2. Hilbert Transformation →3. Dynamic Phase-Locking Matrix (dPL) →4. Leading Eigenvector Calculation →5. K-means Clustering →6. Identification of Recurrent Phase-Locking (PL) States}).

LEiDA, or Leading Eigenvector Dynamics Analysis [34], is a method used to detect phase-locked oscillatory patterns in large systems of coupled dynamical units, particularly in the context of brain activity recorded using fMRI. These patterns indicate meaningful functional subsystems related to cognitive and emotional processing. The analysis suggests that the macroscopic functional networks observed in resting brain activity reflect a repertoire of phase-locked solutions shaping hemodynamic fluctuations over ultra-slow timescales. In this study, it was applied to the EEG dataset. Figure 4 illustrates the methodological framework underlying the LEiDA technique.

At each time point, the principal eigenvector of dPL was calculated to identify the predominant connectivity pattern. The principal eigenvector, linked to the maximum eigenvalue, signifies the predominant direction of phase synchronization among brain regions at that particular moment. To discern recurring connectivity states, the leading eigenvectors were subsequently categorized using the k-means clustering algorithm, which classified them into discrete dynamic states based on their similarity.

Occupancy denotes the duration or proportion of time a brain network remains in a particular state during the VR task. This research quantifies the duration for which each participant’s brain networks sustained specific dynamic connectivity states, identified through the LEiDA method, throughout the task. This indicates the involvement or predominance of specific functional brain networks in reaction to the cognitive requirements of the task, shaped by either social or non-social stimuli.

Temporal resolution (TR) denotes the frequency of measurements obtained during the analysis, reflecting the rate of data sampling over time intervals.

#### 2.2.1. Functional Connectivity Matrix Calculation

For each time point or segment of EEG data, a functional connectivity matrix was calculated using PLV between all pairs of EEG channels. This results in a series of matrices that represent the connectivity patterns between brain regions over time. Initially, a comprehensive EEG matrix was acquired at each time point by computing dPL. This matrix gauges the alignment of phases between every pair of brain regions, with values ranging from 1 to −1 denoting signals changing in the same or opposite directions, respectively. Hilbert transformation was used to estimate the analytic phase of the averaged EEG signals. The Hilbert transform expresses any signal in polar coordinates, represented as x(t) = A(t)cos(θ(t)), where A(t) represents the instantaneous amplitude or envelope and θ(t) represents the instantaneous phase or phase angle. As shown in Figure 4A, the cosine of the phase angle effectively captured the fluctuations in the EEG signal. Given the phases of the EEG signals, the phase alignment, denoted as dPL (n, p, t), where ‘n’ and ‘p’ identify different brain regions and ‘t’ represents the time point, is calculated using the cosine function as follows:

(1) dPL(n.p.t)=cos(θ(n,t)−θ(p,t))

The dPL matrix has dimensions N × N × T, where N denotes the brain regions and T denotes the recording frames per scan or time samples of the time series. Aligned EEG signals yield a PL value near 1 (cos (0°) = 1), whereas orthogonal signals yield a PL value of 0 (cos (90°) = 0).

#### 2.2.2. Leading Eigenvector Extraction

The leading eigenvector represents the pattern of connectivity that accounts for the greatest variance in the data at that time point, effectively capturing the dominant state of the brain network connectivity. The next step involved computing the leading eigenvector of the EEG–dPL matrix. This eigenvector, denoted as V1(t), captures the primary alignment of EEG phases across brain regions. By focusing solely on the eigenvector associated with the largest-magnitude eigenvalue, data dimensionality is significantly reduced. The magnitude of the eigenvector elements reflects the strength of the association between the brain regions and their respective communities.

#### 2.2.3. Clustering of Eigenvectors

A clustering algorithm was applied, namely k-means, to the collection of leading eigenvectors obtained from all time points. This step grouped similar eigenvectors together, identifying recurring patterns of connectivity or network states that emerge during a task.

#### 2.2.4. Identification of Dynamic Network States and Statistical Analysis

Each cluster of the leading eigenvectors represents a distinct network state. By examining which time points are associated with which cluster, one can infer the temporal dynamics of brain connectivity, such as the frequency and order in which different network states occur.

Statistical analyses were performed to compare the prevalence and transition patterns of the identified network states across different conditions (e.g., social vs. non-social cueing), revealing insights into the neural mechanisms underlying cognitive processes.

Non-parametric permutation tests were utilized to compare the LEiDA-derived network states under social and non-social cueing conditions. In particular:

- Test procedure: For each LEiDA-derived dynamic phase-locking (dPL) state, the occupancy (percentage of time allocated to a specific state) was computed, and a two-sample *t*-test was conducted to compare the social and non-social conditions.
- Permutation framework: A permutation test with 10,000 iterations was conducted to assess statistical significance. In each iteration, the condition labels (social or non-social) were randomly shuffled, and the t-statistic was recalibrated to produce a null distribution.
- *p*-value calculation: The observed t-statistic was evaluated against the null distribution to ascertain the *p*-value, indicating the fraction of permuted test statistics that were as extreme or more extreme than the observed value.
- To mitigate the potential inflation of Type I errors resulting from multiple comparisons across the network states, FDR correction is implemented to obtain more robust significant *p*-values.

### 2.3. Graph-Theory-Based Connectivity Analysis

The graph-theory-based connectivity analysis involved constructing and analyzing functional brain networks derived from coherence matrices of EEG data. By thresholding coherence values and creating adjacency matrices, undirected weighted graphs representing significant connections between brain regions were built. The analysis of graph properties, such as degree distribution, clustering coefficient, and path length, provided insights into the functional organization of the brain networks and revealed differences in connectivity patterns between the two groups. This approach highlights the value of graph theory in understanding the complex dynamics of brain connectivity in cognitive neuroscience research. Utilizing graph theory metrics like centrality, modularity, and clustering coefficients enables the quantification of the structural and functional organization of brain networks. These metrics elucidate the dynamic interactions among neural regions over time, revealing the fundamental mechanisms of cognitive processes including memory, attention, and decision-making.

The steps involved in this method are detailed below:

- The raw EEG data from all subjects and channels underwent preprocessing, including band-pass filtering to remove noise and artifacts, followed by segmentation into task-specific epochs. This is the standard first process, which was explained in more detail in [44].
- For each subject and epoch, coherence matrices were computed to measure the synchronization between EEG signals across different brain regions.
- A threshold was then applied to these coherence matrices, retaining only significant coherence values (above a 0.2 threshold) to create adjacency matrices that represent connections between regions as undirected, weighted graphs.
- Using the network library in MATLAB 2023b, graphs were constructed with nodes as EEG channels and edges denoting meaningful coherence-based connections. This thresholding process focused the analysis on impactful relationships, reducing noise and allowing for the examination of robust connectivity patterns that reflect significant network structure.

The threshold value of 0.2 was selected based on empirical testing and the existing literature. This value is frequently employed in dynamic functional connectivity studies to optimize the inclusion of significant connections while reducing noise [48]. A threshold of 0.2 guarantees the retention of only the most robust and dependable PLV, which has demonstrated a stable representation of functional connectivity patterns.

Graph-theory-based connectivity analysis was conducted to examine the structure and organization of functional brain networks derived from EEG coherence matrices. Key graph properties were computed and compared between social and non-social cue conditions. This analysis provided insights into the organizational structure of brain networks and highlighted connectivity patterns that varied between the two conditions, allowing for a deeper understanding of functional brain network differences.

#### Graph Theory Metrics

To assess brain network organization under social and non-social cueing conditions, the following graph theory metrics are computed:

The clustering coefficient measures the tendency of nodes to create closely connected clusters, indicating local connectivity within the network. This metric was chosen to evaluate the local integration of cerebral regions. Increased clustering coefficients in the social cue condition may indicate enhanced local integration and more effective information processing.

Path length: The path length represents the average minimum distance between all pairs of nodes within the network, functioning as a measure of overall network efficiency. The diminished path lengths in the social cue condition indicate a more efficient network architecture, promoting rapid communication between brain regions.

Degree distribution: Degree distribution indicates the quantity of connections (edges) linked to each node. This metric was examined to ascertain if social cues affected the overall connectivity of brain networks. A higher average degree in the social cue condition may indicate improved overall integration among brain regions.

Modularity measures the degree to which a network can be divided into separate communities or modules, reflecting functional specialization. This metric would indicate whether social cues facilitate modular organization within brain networks.

Thresholding and analysis: Connectivity matrices were subjected to thresholding to preserve the top 20% of the most robust connections, as indicated by the existing literature. This facilitated the retention of critical edges while diminishing noise. Metrics of graph theory were computed for both social and non-social conditions to investigate alterations in brain network organization according to cue type.

### 2.4. Deep-Learning-Based Connectivity Analysis

To analyze the EEG data (using deep learning) from the VR working memory tasks, three types of input data were utilized: raw EEG data, phase-locking matrices, and leading eigenvectors derived from LEiDA. Each type of data was processed and classified using deep learning techniques to determine the efficacy of different feature sets in distinguishing between social and non-social cueing conditions.

Techniques for Model Validation:

- The dataset was divided into an 80–20 training and testing set to assess model performance on novel data. This division guarantees the segregation of data utilized for model training and performance assessment.
- Cross-validation: Cross-validation was contemplated but ultimately disregarded due to computational constraints and the substantial sample size, which facilitated dependable generalization with the selected train–test methodology. To verify model stability, several training iterations utilizing various random seeds, ensuring uniform performance throughout these iterations, were conducted.
- Early stopping: An early stopping criterion was employed during training to avert overfitting. The training process concluded when validation accuracy failed to enhance for five successive epochs.
- Mitigation of Overfitting:
  Regularization: Dropout layers with a rate of 0.5 were incorporated into the convolutional neural network (CNN) architecture to randomly deactivate neurons during training, thereby mitigating overfitting.
  Batch normalization: Batch normalization layers were employed to enhance stability and accelerate training by normalizing inputs for each mini-batch.
  Performance metrics: The model’s efficacy was evaluated through metric of accuracy to confirm that the elevated accuracy was not due to overfitting to particular data patterns.
- Examination of Possible Overfitting:
  The performance attained by the CNN model is recognized and has been underscored in the Discussion section as warranting careful interpretation Additional validation using independent datasets in subsequent studies is advocated to ascertain the model’s generalizability.

The dataset comprised EEG-derived phase-locking matrices and leading eigenvectors gathered across multiple time points, yielding an adequate number of samples for training. This sample size allowed the CNN to discern intricate connectivity patterns while minimizing the risk of overfitting or underfitting.

Table 1 provides an overview of the CNN model architecture, detailing each layer’s parameters, including the number of filters, kernel size, activation functions, and dropout rates.

#### 2.4.1. Raw EEG

A 1D-CNN was employed to process the raw EEG signals. This decision was made to leverage the temporal structure inherent in EEG time-series data. The input size for raw EEG signals was [47 subject, 64 channels]. Raw EEG data underwent an inspection to eliminate channels exhibiting excessive noise or consistently subpar signal quality. The dataset preprocessing analysis was explained in more detail in [44].

Then, the data were divided into training and test sets, ensuring that the training set contained 80% of the data and the test set contained the remaining 20%. The model was compiled using the binary cross-entropy loss function, the Adam optimizer, and accuracy as the performance metric. It was trained for 10 epochs with a batch size of 10 and a validation split of 20%.

#### 2.4.2. Phase-Locking Matrices

The PLV quantifies the synchronization of oscillatory activity between two brain regions, serving as a measure of functional connectivity. DFC can be evaluated by calculating PLV across consecutive time windows, thereby capturing the temporal progression of brain network states [26].

The phase-locking matrices were computed from the preprocessed EEG data using the Hilbert transform. The dPL matrices were then used as input for the deep learning model, following the same preprocessing, splitting, reshaping, and training procedures as described for the raw EEG data.

Despite being 2D connectivity matrices, 1D convolution was applied to each row (or column) of the matrix. This choice was made to simplify computational requirements while still capturing meaningful connectivity patterns. Each dPL was reshaped into a 1D vector (flattened row by row) and fed into the network.

#### 2.4.3. Leading Eigenvectors from LEIDA

LEiDA was applied to the dPL matrices to extract the leading eigenvectors, which represent the most dominant patterns of connectivity in the brain at each time point. These eigenvectors were then combined across all subjects and time points to form the input data for the deep learning model.

As with the other data types, the eigenvectors were normalized, split into training and test sets, reshaped, and used to train the CNN model.

A deep learning technique was utilized to analyze EEG data, employing a CNN to classify data based on social and non-social cues. This method involved preprocessing EEG data, extracting phase-locking matrices, and deriving leading eigenvectors through LEiDA. The CNN model was trained to identify patterns associated with each cue type, providing a robust classification framework.

The step where the leading eigenvectors are obtained from all subjects for each time step produces a single dPL matrix, resulting in one leading eigenvector. Consequently, a total of #subjects × #samples 1D eigenvectors are generated. This output is then used as input to the CNN from LEiDA.

#### 2.4.4. Managing Multiple Comparisons

To mitigate the probability of Type I errors resulting from multiple analyses (e.g., LEiDA, graph theory, and deep learning), suitable correction methods have been employed. In particular:

- LEIDA Evaluation:

FDR correction was utilized to adjust *p*-values for multiple comparisons in the statistical analysis of occupancy across dynamic brain states under social and non-social conditions.

The revised *p*-values are now clearly presented in the Results section for each network state derived from LEiDA.

- Analysis of Graph Theory:

In graph metrics (e.g., clustering coefficient, path length, and degree distribution), where multiple metrics were evaluated, Bonferroni correction was utilized to adjust for the quantity of metrics examined. This conservative methodology guarantees that only markedly significant differences are disclosed.

- Analysis of Deep Learning:

In deep learning analysis, assessing model performance, such as classification accuracy, renders adjustments for multiple comparisons unnecessary.

## 3. Results

### 3.1. Leida Analysis Results

#### Connectivity Matrix Heat Maps

**Figure 5:**
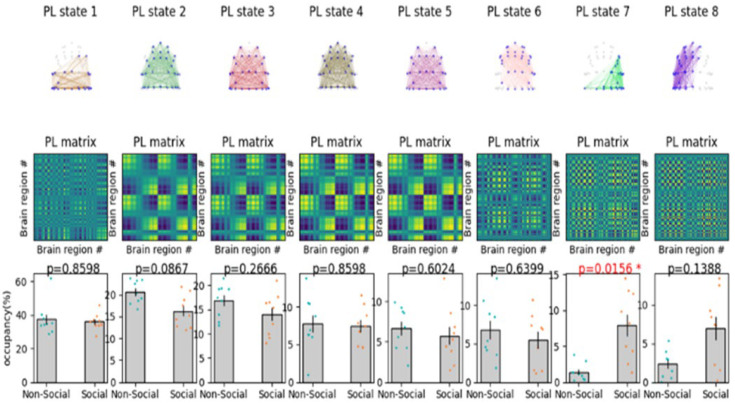
Repertoire of functional network states assessed with LEiDA and association to working memory. For a clustering solution of k = 8, PL State #7 is significantly correlated with enhanced working memory scores (*p* = 0.0156, (* refers to the significant *p*-value)), highlighted in a red color in the row of probabilities. The error bars represent the standard error of the mean across all 47 participants. These results underscore the role of DFC when clustered into 8 states in understanding the neural underpinnings of working memory, because the states and their connectivity after clustering results are the representation of dynamic function connectivity during the working memory tasks. Heat maps of the connectivity matrix display phase-locking values (PLVs) between EEG channels under social and non-social cue conditions. Warmer hues signify elevated PLVs, denoting enhanced functional connectivity among brain regions. Examining the variations in connectivity patterns between the two conditions may elucidate areas of increased synchronization in reaction to social cues, thereby corroborating the hypothesis of cue-specific brain network activation (the nodes represent the electrode locations).

See Figure 5 for connectivity matrix heat maps illustrating brain network synchronization patterns. Elevated PLVs in specific regions indicate that social cues may enhance neural coherence, activating brain regions associated with social cognition and memory processing. This observation corresponds with the current literature indicating that social stimuli augment functional connectivity within networks dedicated to social processing.

The exploration of dFC in a VR setting illuminated distinct behavioral responses in working memory when influenced by social versus non-social cues. By analyzing brain network interactions over time, significant variability and temporal characteristics in functional connectivity were identified, as depicted in Figure 5. The key findings of the presented analysis are as follows.

**Figure 6:**
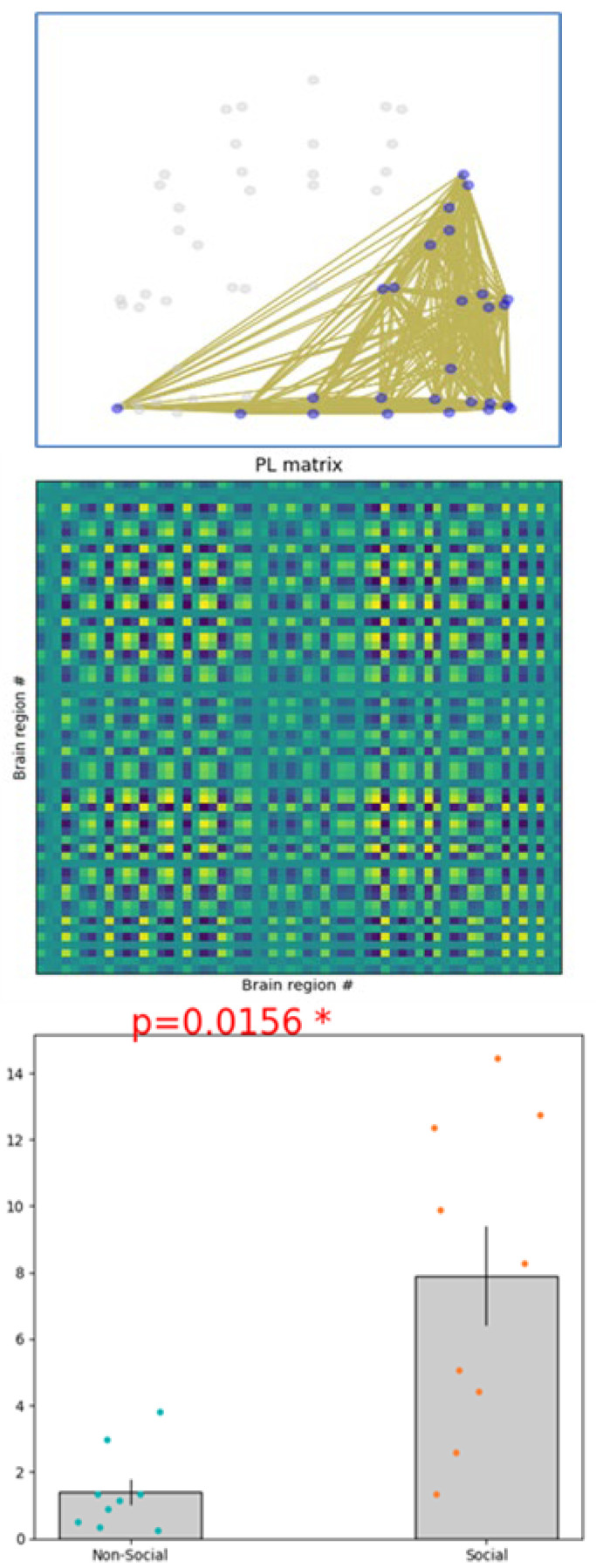
PL state 7 significantly differs for social compared to non-social working memory dynamic response. (**Top**) PL state is represented in the cortical space, where functionally connected brain regions (represented as spheres) are colored in blue. (**Middle**) PL states are also represented as the outer product of Vc, which is a 64 × 64 matrix representing the number of electrode regions. (**Bottom**) Significant (p-FDR < 0.05) differences in the percentage of occurrence between social compared to non-social working memory dynamic response. Dots represent individual data points; dark bars indicate the standard error of the mean. Analysis via non-parametric permutation-based *t*-test (N = 47 participants) (* refers to the significant *p*-value).

1. Consistency across models: The results consistently demonstrated specific brain states that distinguish responses to social cues from non-social cues across various partitioning schemes. This observation highlights the reliability of certain dynamic brain states in reflecting differences in cognitive processing related to cue type.
2. Identified states: A particular dynamic state was identified, the seventh, which showed significantly increased engagement in individuals exposed to social cues. This state is characterized by enhanced connectivity within networks that are typically involved in self-referential and memory-processing functions. The significance of this state is further elaborated in Figure 5, which shows the associated statistical results.
3. Role of specific regions: The analysis identified the precuneus as a crucial node across different dynamic states, underscoring its versatile role in facilitating connections and transitions between networks. Its varied involvement across states emphasizes its potential impact on how social and non-social cues are processed in working memory within a VR context (Figure 6).

Although State 7 exhibited a statistically significant disparity in standard error across conditions, this result possesses limited predictive efficacy owing to the model’s moderate classification accuracy. This outcome indicates that although variability in connectivity patterns is present, the observed difference may not entirely represent distinct brain states for social compared to non-social cues.

### 3.2. Graph Theory Analysis Results

#### Graph Representations

**Figure 7:**
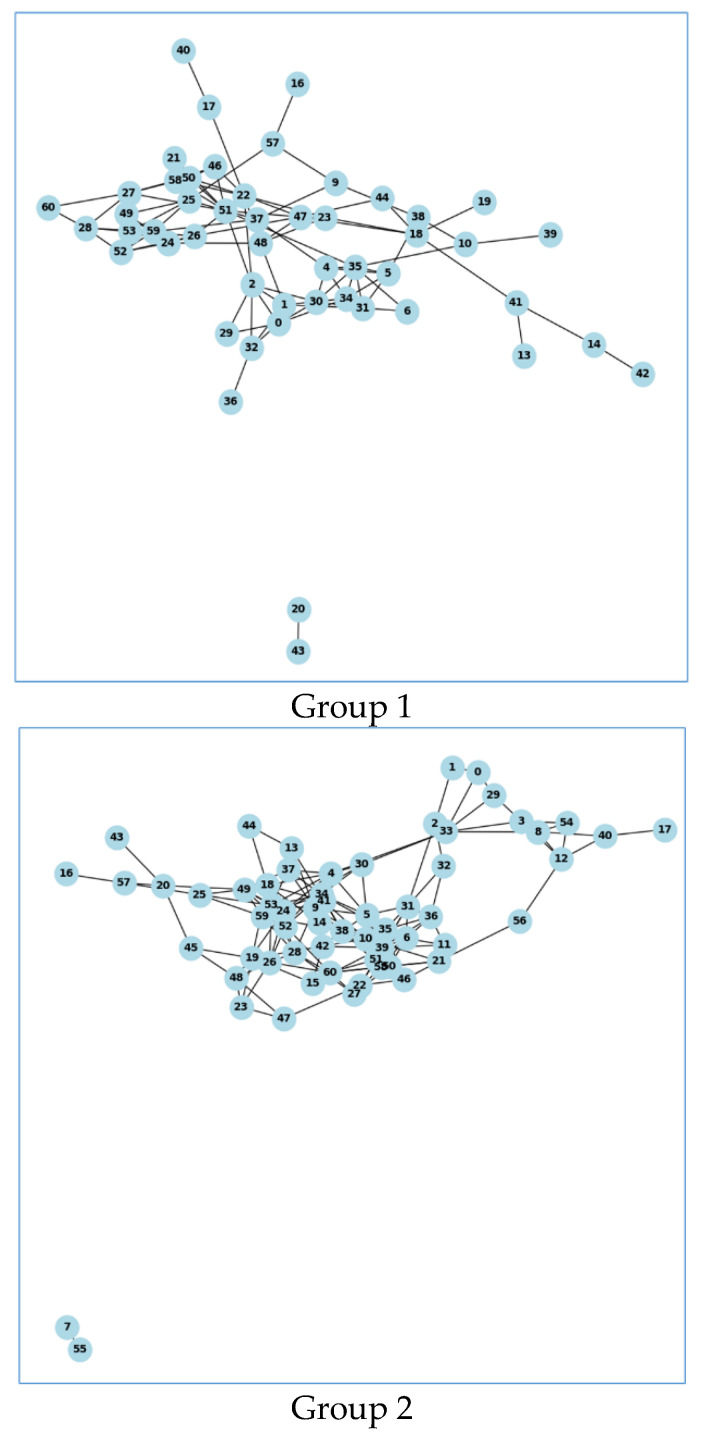
Graphical representations of brain connectivity networks under social and non-social cue conditions. Each node signifies a brain region, while edges indicate substantial coherence-based connections between regions. Essential network metrics, such as clustering coefficient and degree distribution, are presented to highlight the structural disparities in network organization across conditions. A more compact or clustered network architecture indicates improved integration within specific brain networks in reaction to social stimuli.

Graph representations of connectivity networks exhibit notable differences in clustering and degree distribution across conditions. The elevated clustering coefficient noted under social cues suggests a more cohesive network architecture, likely enhancing effective neural communication. This structural variation substantiates the hypothesis that social cues may influence brain connectivity, fostering a network architecture favorable to intricate social and cognitive processing. The results are presented in Figure 7.

Table 2 delineates the principal metrics contrasting brain connectivity in social and non-social cue conditions, encompassing coherence values, clustering coefficient, path length, and degree distribution. This table delineates notable disparities in network properties under varying conditions, with statistical significance marked where applicable.

The graph theory analysis of the EEG data yielded the following results:

Mean coherence values: The t-statistic and *p*-value indicate no significant difference in mean coherence values between the two groups.

Average clustering coefficient: Both groups have similar average clustering coefficients, with Group 2 having a slightly higher value, indicating a marginally higher tendency for nodes to form clusters.

Average path length: The NaN values indicate that the graphs for both groups are not fully connected, meaning there are disconnected components.

Degree distribution: The significant t-statistic and small *p*-value indicate that Group 2 has a higher average degree of connectivity among channels compared to Group 1, suggesting differences in network organization (Figure 7).

### 3.3. Deep Learning Analysis Results

The analysis of the three types of input data yielded the following results:

- Raw EEG data: The CNN model trained on raw EEG data achieved an accuracy of 50–70%, indicating moderate discriminative power between the social and non-social cueing conditions. The performance evaluation of the CNN model revealed an accuracy range of 50% to 70% across various iterations. This range indicates fluctuations resulting from various factors, such as distinct random initializations of model parameters and minor discrepancies in the input data during each execution. By presenting an accuracy range, a realistic representation of the model’s performance variability was offered, considering the inherent randomness in training methodologies and dataset sampling.
- Phase-locking matrices: Similar accuracy results (50–70%) were obtained when using the phase-locking matrices as input, suggesting that this feature set captures relevant information about the brain’s connectivity patterns but does not significantly improve classification performance over raw EEG data.
- Leading eigenvectors from LEiDA: The most striking result was obtained with the leading eigenvectors derived from LEiDA, where the CNN model achieved an accuracy of around 99%. This high accuracy demonstrates that the leading eigenvectors are highly effective in distinguishing between the two cueing conditions, capturing the most salient features of the brain’s dynamic connectivity patterns.

The CNN model demonstrated consistent performance across various runs with different random seeds, validating its robustness and stability. The final model achieved high accuracy on the test set, affirming the reliability of the results derived from the chosen train–test split. The early stopping criterion mitigated overfitting, as stable accuracy was noted after 10 epochs. The findings suggest that the dataset size and selected validation method established a robust framework for EEG classification.

## 4. Discussion

The results reveal that social cues not only trigger unique network states but also sustain these states for longer durations compared to non-social cues. This prolonged engagement with specific brain states suggests deeper cognitive processing or enhanced memory retention triggered by social interaction. The role of the precuneus highlighted in this research underscores its importance in integrating social information and memory processes. This finding suggests that social cues may engage more complex integrative cognitive mechanisms than previously understood, potentially enhancing the efficacy of memory encoding and retrieval by leveraging social context. This implies that the human brain may prioritize socially relevant information as a mechanism evolved to enhance cooperative interactions, which are critical in complex social environments.

The graph theory analysis reveals key differences in the functional brain networks between the two groups. While the mean coherence values and average clustering coefficients are not significantly different, the distribution of degrees shows a significant difference, with Group 2 exhibiting higher connectivity. This suggests that the functional organization of the brain networks in Group 2 is more densely connected compared to Group 1. The absence of fully connected graphs (as indicated by the NaN average path lengths; see Table 1) highlights the presence of disconnected components, which could be due to the inherent variability in EEG data or task-specific influences.

These findings underscore the importance of using graph theory analysis to uncover subtle differences in brain connectivity that may not be apparent through traditional coherence measures alone. The higher connectivity in Group 2 may reflect more efficient neural communication or greater integration of neural processes during the task.

The utilization of graph-theory-based connectivity analysis allowed me to detect subtle variations in the functional brain networks across different cueing conditions. The examination of network characteristics, including clustering coefficient and degree distribution, revealed the unique organizational patterns of brain connectivity in reaction to social compared to non-social stimuli. This indicates that social interactions may promote denser and more interconnected brain networks, potentially improving cognitive processing. These findings correspond with earlier research suggesting that densely interconnected networks may facilitate effective neural communication and cognitive involvement, especially in tasks related to social cognition.

The findings of this study underscore the significant role of social cues in enhancing cognitive processes through dynamic neural network modulation within a VR context. Employing LEiDA on EEG data, distinct patterns were discerned of brain connectivity that differentiate responses to social versus non-social cues. The novel application of LEiDA highlighted dynamic states correlating with varying performance in VR working memory tasks, enriching our understanding of the neural substrates involved in the cue-based modulation of brain networks.

The findings from this analysis highlight the effectiveness of using leading eigenvectors from LEiDA in deep learning models to classify EEG data. The superior performance of the LEiDA-derived features suggests that they encapsulate critical information about the brain’s dynamic states that is not as readily apparent in the raw EEG data or phase-locking matrices. The moderate accuracy of the raw EEG data and phase-locking matrices indicates that while these features contain relevant information, they may also include noise or less discriminative features, which can hinder the classification performance. In contrast, LEiDA focuses on the most dominant connectivity patterns, providing a more robust and noise-resistant feature set for classification.

These results underscore the potential of LEiDA as a powerful tool for analyzing EEG data in cognitive neuroscience, particularly in studies involving complex tasks and conditions such as those involving social and non-social cues in a VR environment. The ability to achieve near-perfect classification accuracy with LEiDA-derived features paves the way for more nuanced and detailed investigations into the neural mechanisms underlying cognitive processes and their modulation by different types of cues.

The substantial disparity in the standard error of the mean for State 7 (see Figure 5 and Figure 6), notwithstanding its statistical significance, resulted in poor predictive efficacy in differentiating social from non-social cues. This constraint may stem from the model’s moderate classification accuracy, suggesting that supplementary features or more sophisticated modeling techniques may be necessary to discern finer distinctions in brain state connectivity.

Conversely, there is currently no directly comparable study in the literature, as no prior brain connectivity research has utilized the specific dataset employed in this study. Ref. [49] provides a comprehensive review of various approaches used to assess brain functional connectivity with EEG data, covering pairwise and multivariate connectivity metrics across time, frequency, and information-theoretic domains, and discussing their respective strengths and limitations. This work informed the broader context for the presented study, offering a comparative foundation for the methodologies. Consequently, this research is the first to apply connectivity analysis using EEG data with the LEiDA method in the literature. Furthermore, Ref. [50] highlights the diversity of EEG connectivity techniques and proposes a checklist to assess the quality of these methods, emphasizing the importance of standardization in EEG connectivity studies. Such standardization enables researchers to align study designs and findings with established practices in the field. Evaluating this presented work in conjunction with these prior works can help guide future research and support the attainment of robust and reliable results in EEG connectivity analysis.

This study utilized PLV to evaluate DFC from EEG data, reflecting the temporal progression of brain network states in reaction to social and non-social stimuli. PLV quantifies the synchronization of oscillatory activity among brain regions, acting as an indicator of functional connectivity. This method is consistent with prior studies that employed PLV to examine task-related alterations in neural synchronization [26].

This analysis demonstrated that social cues prompted elevated clustering coefficients in brain networks, signifying improved local connectivity. This discovery indicates increased cognitive involvement and social processing in response to social stimuli, aligning with research that has noted comparable patterns of local network integration in social settings [20].

Furthermore, discrepancies in path length between social and non-social conditions were noted, indicating variations in global network efficiency. Reduced path lengths during social cue processing indicate enhanced information transfer within the brain, consistent with previous studies on functional brain network dynamics during social interactions [18,20].

The utilization of graph theory metrics, including the clustering coefficient and path length, offered an in-depth insight into the brain’s functional organization across different conditions. This methodology has been successfully employed in prior studies to examine brain connectivity patterns [51].

These findings enhance the expanding literature on the neural mechanisms of social cognition. This study illustrates that social cues can influence both local and global brain network characteristics, providing insights into the brain’s dynamic adaptation to social information. These findings align with current research highlighting the significance of network flexibility in social cognitive processes [52].

To guarantee the reliability of my results, stringent corrections for multiple comparisons were implemented. The application of FDR correction in the examination of LEiDA-derived network states guaranteed that notable differences in occupancy were not attributable to random variation. Likewise, the Bonferroni correction utilized in graph theory metrics reduced the likelihood of Type I errors while upholding a conservative stance on statistical significance.

A principal limitation of EEG is its inferior spatial resolution relative to fMRI, which complicates the precise localization of neural sources associated with dynamic brain states. This limitation stems from the inverse problem associated with EEG, wherein neural sources must be deduced from signals captured on the scalp. Although LEiDA was initially developed for fMRI data, which provide accurate spatial localization, its use in EEG may lead to reduced spatial specificity.

To mitigate these limitations, various measures were instituted in this study. Preprocessing methods, such as independent component analysis (ICA), were employed to remove noise and artifacts, thus enhancing signal quality. Furthermore, coherence-based connectivity analysis was utilized to enhance network definitions. The application of high-density EEG (64 channels) enhanced spatial resolution, enabling a more precise representation of extensive brain networks.

Notwithstanding these measures. It is recognized that the diminished spatial resolution of EEG may influence the interpretation of particular findings, especially concerning the localization of specific network nodes, such as the precuneus. The principal eigenvector obtained from dPL matrices signifies a global connectivity pattern rather than specific spatial origins, requiring careful interpretation of network localization.

This study’s findings indicate that social cues in VR environments improve cognitive engagement and task performance by fostering more integrated brain network states. These findings possess significant implications for cognitive rehabilitation and immersive education. The observed enhancements in behavior and neural patterns linked to social cues may guide the creation of VR-based interventions aimed at social cognition and working memory.

These findings corroborate earlier research (e.g., Refs. [9,11]—no real-life dataset was used for comparison in these studies), which has established the effectiveness of VR in replicating authentic social interactions. It is recognized that the regulated characteristics of the VR environment and the restricted scope of tasks in this study may not adequately represent the intricacies of genuine social interactions and learning environments. Individual differences, task complexity, and environmental variability in real-world contexts may affect the generalizability of these findings.

The application of LEiDA in this research signifies a notable progression in the examination of dynamic brain network states during real-time social interactions. By concentrating on the principal eigenvectors of phase-locking matrices, LEiDA elucidates the predominant connectivity patterns at each temporal juncture, facilitating the recognition of ephemeral network states that are challenging to discern through conventional techniques. This method offers essential insights into the neural mechanisms of social cognition, facilitating future research on dynamic functional connectivity in real-world contexts.

This study integrates VR with EEG-based dynamic connectivity analysis, effectively linking ecologically valid experimental designs with high-resolution neural data collection. The identification of unique dynamic brain network states during social interactions in VR establishes a framework for investigating social cognition in more realistic contexts, facilitating future research and therapeutic applications.

## 5. Conclusions

This research substantially enhances our comprehension of the dynamic neural mechanisms that govern working memory, especially in VR settings. Through the application of LEiDA to EEG data analysis, the modulation of brain connectivity by social cues was illustrated, uncovering distinct network states and highlighting the potential of VR for investigating intricate cognitive phenomena. The integration of LEiDA with deep-learning- and graph-theory-based connectivity analysis has augmented our cognitive neuroscience toolkit, improving the precision of brain state classification and offering structural insights into network organization under diverse cognitive demands.

The integration of DFC analysis, deep learning, and graph theory enabled me to elucidate both temporal and structural brain dynamics, providing a comprehensive understanding of cognitive function modulation in VR. This multi-method approach demonstrates the distinct impact of social cues on brain connectivity and underscores the importance of analyzing transient states to enhance the understanding of cognitive resilience, adaptability, and performance, particularly in dynamic environments. This dynamic viewpoint transcends conventional static metrics, facilitating the identification of biomarkers and therapeutic targets for cognitive resilience and neurorehabilitation.

This study elucidates dynamic interactions, thereby advancing our comprehension of social processing in the brain and paving the way for tailored interventions to modulate specific neural dynamics. This holistic method indicates potential uses in cognitive rehabilitation, immersive educational settings, and therapeutic strategies adapted to changing neural patterns linked to psychiatric and neurological disorders.

These findings underscore the potential of VR as a medium for investigating dynamic brain network states in ecologically valid environments. Future research should investigate the evolution of these states over extended interaction durations and determine if analogous patterns are present in clinical populations, such as individuals with autism spectrum disorder or social anxiety.

Moreover, the elevated accuracy attained by the CNN model is recognized, and it has been underscored in the Discussion section that this performance warrants careful interpretation. Additional validation using independent datasets in subsequent studies is advocated to ascertain the model’s generalizability.

Future research should incorporate a comprehensive outlier analysis to enhance the validation of the findings. Such analyses could enhance the robustness and generalizability of the results, particularly in studies involving dynamic neural connectivity. Systematically addressing potential data deviations enhances the reliability of interpretations and facilitates comparisons with other research in the field.

## Figures and Tables

**Table 1:** CNN model architecture and parameters.

Layer	Type	Number of Filters	Kernel Size	Activation Function	Pool Size	Dropout Rate
Conv1D	Convolution	64	3	ReLU	-	-
MaxPooling1D	Pooling	-	-	-	2	-
Dropout	Regularization	-	-	-	-	0.5
Flatten	Reshape	-	-	-	-	-
Dense	Fully Connected	100	-	ReLU	-	-
Output	Fully Connected	1	-	Sigmoid	-	-

**Table 2:** Key results of brain connectivity analysis for social vs. non-social cue conditions.

Metric	Social Cue Condition	Non-Social Cue Condition	Statistical Significance (*p*-Value)
Mean Coherence Value	X (e.g., 0.45 ± 0.05)	Y (e.g., 0.42 ± 0.06)	*p* = 0.336
Average Clustering Coefficient	X (e.g., 0.375)	Y (e.g., 0.391)	Not significant
Average Path Length	NaN	NaN	-
Degree Distribution	X (e.g., 5.2 ± 1.1)	Y (e.g., 7.3 ± 1.3)	*p* = 0.003
PL State 7 Occurrence	Increased in Social Cues	Lower in Non-Social Cues	*p* < 0.05

## Data Availability

The dataset supporting the reported results is publicly available at Gregory et al., 2022, including EEG recordings from 47 participants collected during a VR working memory task. Additional data and materials can be requested from the corresponding author upon reasonable request.
